# Life Quality in Premenopausal Women after Embolization of Uterine Myomas

**DOI:** 10.3390/jpm12121990

**Published:** 2022-12-01

**Authors:** Panagiotis Tsikouras, Foteini Gkaitatzi, Aggeliki Gerede, Xanthoula Anthoulaki, Anastasia Bothou, Anna Chalkidou, Spyridon Michalopoulos, Ioannis Tsirkas, Selma Gyroglou, Panagiotis Peitsidis, Konstantinos Nikolettos, Alexios Alexiou, George Dragoutsos, Natalia Sachnova, Pelagia Chloropoulou, Stefanos Zervoudis, George Iatrakis, Werner Rath, Grigorios Trypsiannis, Nikolaos Nikolettos, Vasileios Souftas

**Affiliations:** 1 Department of Obstetrics and Gynecology, Democritus University of Thrace, 68100 Alexandropoulis, Greece; 2 Rea Maternity Hospital, University of West Attica, 17564 Athens, Greece; 3 Department of Anaesthesiology, Democritus University of Thrace, 68100 Alexandroupolis, Greece; 4 Department of Interventional Radiology, Obstetrics and Gynecology, Democritus University of Thrace, 68100 Alexandroupolis, Greece

**Keywords:** premenopausal women, uterine fibroids, uterine artery embolization, life quality

## Abstract

**Objectives:** Fibroids cause significant morbidity and are the most common indication for hysterectomies worldwide, delimiting a major public health problem. Uterine artery embolization (UAE) is an alternative therapy to surgical treatment of symptomatic fibroids; it has satisfactory long-time results and is no longer considered investigational for the treatment of symptomatic fibroids. This study was undertaken to evaluate changes in fibroid specific symptom severity and health-related quality of life (HRQOL) after UAE and to optimize the assessment of safety and outcomes measures for participants who receive UAE to objective compare UAE and surgical alternatives for therapy of symptomatic fibroids. **Study design:** The analysis was based on questionnaires completed by 270 pre-menopausal females with a mean age of 42 years (range, 38–50 years) who underwent UAE for uterine leiomyomas and/or adenomyosis from November 2013 through December 2019. Only symptomatic women were selected whose symptoms were not improving with medication and who did not wish to have children. The primary outcome measure was a change in fibroid symptoms and HRQOL (health related quality of life) after UAE. Secondary outcomes included the decrease in uterine volume after UAE. **Results:** Questionnaires were completed by 270 women (100%) at a mean of 12.1 months from UAE. The median follow-up period was two years. Uterine fibroid embolization led to a shrinkage at three months for the 90% of the participants. A reduction of bleeding symptoms, pain and bulk-related symptoms was observed in 89.7%, 88.9%, and 89.5% of the patients, respectively. In the long term, there was no significant difference in parameters assessed compared with the midterm follow-up findings. A total of 6 patients (2.3%) underwent fractional curettage an average of 32.1 months after intervention due to necrotic changes in submucosal fibroids. All participants continued to be satisfied with the intervention, and 240 patients (88.9%) answered that they would recommend uterine fibroid embolization to other patients. **Conclusions:** Women who undergo UAE have a significant decrease in symptom severity and increase in HRQOL which is associated with high levels of satisfaction with the procedure (even when subsequent therapies are pursued).

## 1. Introduction

Fibroids are benign tumors of the uterus which originate from myometrial cells and during their development repel surrounding tissues. This is the most common disease of the female reproductive system, with an incidence of 70–80% by the age of 50 [1,2]. Fibroids are more common in women aged 30–40 years old. It is estimated that 20–40% of women will develop fibroids within this decade [1,2]. The majority of fibroids do not cause symptoms in women. Problems begin when the fibroids exceed a certain size or their position disrupts the function of the uterus. In some cases, especially if their size exceeds more than 10 cm, large fibroids can cause pressure on the bladder and ureters, leading to frequent or difficult urination and even hydronephrosis, and also on the rectum, leading to constipation or tension [1,2,3]. The fact that fibroids are more likely to develop during reproductive age sometimes causes problems in family planning. Previously, their treatment usually involved their removal or even hysterectomy. Fibroids need to be treated when they cause symptoms such as menorrhagia or press the surrounding organs, when they affect woman’s fertility or put a future pregnancy at risk, and when malignancy is suspected [4,5,6,7,8,9]. In the absence of the above, regular monitoring is recommended. The medical treatment of fibroids focuses on relief of symptoms and not in their treatment as the fact that they cannot be eliminated without surgery. The action of GnRH analogs centrally in the brain causes artificial menopause which leads to a decrease in estrogen levels which in turn leads to shrinkage of fibroids [4,5,6,7,8,9]. This treatment is not a permanent solution, as this prolonged hypoestrogenic condition causes menopausal symptoms and carries a risk of bone loss. Also, discontinuation of treatment for two to three months leads to an increase in estrogen levels resulting in fibroids regrowing. The analogs are usually given either premenopausally for a few months until menopause occurs naturally or as a preoperative treatment to control bleeding and shrink fibroids, which will facilitate surgical access [4,5,6,7,8,9].

The exact cause of fibroids remains unknown. However, predisposing factors have been identified, the main ones of which are:

(1) Ethnicity: the incidence of fibroids is significantly higher in black women.
(2) Age: fibroids are not found before puberty [10,11,12,13,14].
(3) Genetic predisposition: women with first degree relatives with fibroids are at greater risk of appearing themselves [15,16,17,18].
(4) The hormonal state of the woman: early menopause increases the risk of fibroids, while delayed menopause seems to act protectively. In addition, during pregnancy, where estrogen production increases, some women experience an increase in fibroid size during the first trimester. Similarly, after menopause (when estrogen levels fall), fibroids regress. In women receiving hormonal replacement therapy, they usually increase in size. However, women with fibroids do not appear to have higher levels of estrogen in their blood. In conclusion, estrogen seems to promote the growth of fibroids, but not to be responsible for their appearance [19,20].
(5) Body weight: the high body mass index (BMI), as well as the consumption of large amounts of red meat seem to contribute to the development of fibroids [19,20]. Protective factors are breastfeeding, high consumption of fruits and vegetables, etc.
(6) Hypertension is clearly associated with fibroids: it has been suggested that atherosclerotic damage to the blood vessels of the uterus and the subsequent inflammatory condition may play a role in the development of fibroids. Additional blood pressure-related endocrine factors such as angiotensin II are suspected of proliferating fibroids through the angiotensin II type 1 receptor [19,20].

Concerning genetic predisposing factors, several epidemiological studies have shown a significant genetic effect, especially in cases where leiomyoma manifest at a young age. The patient’s first-degree relatives have a 2.5-fold increased risk of developing the disease, while this risk increases by six times in cases of early-onset disease. Monozygotic twins are twice as likely to have a hysterectomy as monozygotic twins. Fibroids are monoclonal benign tumors, of which 40–50% show chromosomal abnormalities that can be detected in the karyotype. In the case of multiple fibroids, they are usually caused by unrelated genetic defects. Specific mutations in the MED12 gene have been implicated in 70% of fibroids [15,16,17,18,19,20,21,22].

Also, a fibroid’s growth is mainly affected by hormones (specifically from estrogen and progesterone). Both are believed to have mitogenic effects on leiomyoma cells and also act by affecting a large number of growth factors, cytokines, apoptotic agents and other hormones. In addition, the actions of estrogen and progesterone are regulated by a cross-talk between estrogen, progesterone, and prolactin the signals of which control the expression of the respective nuclear receptors [15,16,17,18,19,20,21,22]. It is believed that estrogens promote the growth of fibroids by increasing the growth factors IGF-1, EGFR, TGF-beta1, TGF-beta3, and PDGF and contribute to the abnormal survival of leiomyoma cells by reducing the growth factor 5 of the anti-apoptotic agent PCP4 and the PPAR-gamma signaling competition. Progesterone is believed to promote fibroid growth by increasing EGF, TGF-beta1 and TGF-beta3, while surviving by increasing Bcl-2 expression and decreasing TNF-alpha [15,16,17,18,19,20,21,22]. While in pre-menopausal fibroids, ER-beta, ER-alpha, and progesterone receptors are overexpressed, in rare post-menopausal fibroids only ER-beta receptors are found overexpressed. The majority of studies have concluded that polymorphisms in the ER and PR gene are not associated with the occurrence of fibroids in Caucasian populations. Nevertheless, a specific ER-alpha genotype was found to be associated with the appearance and size of fibroids. The increased prevalence of this genotype in black women may thus explain the increased presence of fibroids in this population group [15,16,17,18,19,20,21,22].

On the other hand, elevated estrogen levels can result from conditions such as pregnancy, use of contraceptive pills and exogenously administered estrogens and are observed during a woman’s reproductive age (15–45 years). Additional factors include polycystic ovary syndrome, diabetes, and hypertension.

Many women consider hysterectomy to be an amputation of their femininity and sexuality and look for alternatives, although studies have shown an improvement in quality of life after hysterectomy [10,11,12].

Women with fibroids have compromised overall quality of life and impairment in many specific domains including work productivity, self-image, relationships, and social, emotional and physical well-being [23].

The aim of this study was to investigate the effect of embolism on the quality of life of premenopausal women with fibroids, particularly on the reduction of pain and stressful symptoms (and moreover on the reduction of bleeding).

## 2. Material and Methods

270 pre menopausal females who underwent UAE for symptomatic uterine leiomyomas and/or adenomyosis were included in the study. The mean age of the patients was 42 years (range, 38–50 years). Ethical approval for this procedure was confirmed by the ethics committee of the University Hospital in Alexandroupolis, Democritus University of Thrace (Alexandroupolis, Greece; reference no. 8/37–10/10/13). Selection criteria for the patients were: (1) to be symptomatic; (2) to have symptoms that did not subside with medication; (3); to have had diagnosis of the condition made with transvaginal ultrasound and/or MRI’ (4) to have a urinary tract infection excluded; (5) to be investigated with a thorough laboratory-hormonal test including regular blood testing, FSH, LH, AMH, estrogen and progesterone (with “normal” values for the age); (6) not wishing to have children. The reason that women with a desire to have children were not included in the study based on the deficit of knowledge of the participants about this method and due to this fact had luck of trust. The relevant symptoms of the symptomatic participants included menometrorrhagia, dysmenorrhea, dyspareunia, and symptoms attributed to bulky disease or pressure on pelvic organs. Exclusion criteria were referred to post menopausal females, patients with serious comorbidities, patients wishing to preserve their fertility, patients with known allergy to the contrast agent utilized during the procedure and patients with a suspected malignant condition. The women were screened with imaging and endometrial biopsy to rule out malignancy. The most important symptoms, as was clear from the medical history, included menometrorrhagia, dysmenorrhea, dyspareunia, symptoms attributed to bulky disease or pressure on pelvic organs, and reduced sexually activity. Large fibroids (>15 cm) were expected to shrink, but their final size may again cause compressive symptoms, therefore they were a relative contraindication nevertheless were included in the study. Other conditions to consider were the presence of submucosal or polypoid fibroids, which are best excluded endoscopically as their necrosis can lead to miscarriage with late bleeding and pain.

The coexistence of an active infection that could recur with ischemia, a history of radiotherapy that has already reduced uterine perfusion, and renal failure due to the toxic effect of the shader were considered contraindications. The preoperative imaging examination included the obligatory MRI scan (to look for signs of malignancy), while MRI scans were repeated in the 1st, 3rd, 6th and 12th month, to monitor the course of the disease and the consequences of the operation. At the same time, women were evaluated according to clinical, imaging, laboratory and hormonal examinations (regular blood testing, FSH, LH, AMH, estrogen, and progesterone).

### 2.1. Technique of UAE

After percutaneous puncture of the common femoral artery, angiography was performed by injecting a contrast agent into the abdominal aorta shortly before its division. This was followed by selective unilateral catheterization of the left uterine artery with a special thin 4-F catheter and advancing this catheter to the uterus as far as possible. A flexible 2.7-F microcatheter was then advanced through the lumen of this catheter to the carrier artery of the fibroid. Following the promotion of the embolospheres (diameter 500–900 μm)under continuous radioscopic examination to avoid embolization of the healthy myometrium or other unwanted embolism, the elimination of the vascular fibroid was checked. In the majority of the patients, a 2.7 or 2.8 microcatheter was used to access the uterus following selective catheterization of the uterine artery. Hydrogel coatedacrylic microspheres 500 or 700 μm in size were used as an embolic agent in cases of adenomyosis, and the same microspheres, sized 700 and 900 μm, were administered slowly in cases with myomas. The right uterine artery was initially selectively accessed with the crossover technique and, when the catheterbypassed the arteries supplying the vagina and cervix, administration of the embolizing particles was initiated. Then, the same approach followed also for the left uterine artery. In all cases, antibiotics were also administered during the intervention, by intravenous injection. Clinical, laboratory and imaging follow up examinations by transvaginal ultrasonography and MRI scans were performed in the first, third, sixth, ninth and twelfth months after the procedure. Heparin prophylaxis due to immediate patients’ mobilization was not necessary and not recommended according to international guidelines. Post-UAE was compared with the size prior to treatment.

### 2.2. Questionaire

The quality of life (QoL) was assessed by an anonymous questionnaire, originally created for the purposes of the study. Available validated tools of QoL were not used in this study to avoid narrowing fields of interest. As an example, we did not use the related questionnaire of Spies et al. [24] and Bucek et al. [25] since it is obvious that urination during night hours (nocturia) can seriously affect quality of life, a specific question not included in the previous studies. Questions included in the questionnaire were tested for comprehension in 10 patients and modified (clarified) in one question. Changes in the size of fibroids were assessed by volume, while the qualitative characteristics of their degeneration were studied. The effects of the method on the menstrual cycle of women, the laboratory-hormonal values and, in the long run, the effects of the method on fertility were studied. In particular, the questionnaires included the following questions:

(1) Has there been bleeding during menstruation for at least six months and a description of the bleeding after the embolism?
(2) Were passing blood clots reported during menstruation for at least six months after embolization?
(3) Were there any reduced or unchanged symptoms (flatulence, pelvic floor heaviness, frequent urination during the day) after the embolization?
(4) Did frequent urination during the nighttime hours decrease or remained unchanged after embolization?
(5) How do you describe the fluctuation during the menstrual cycle after embolization compared to previous cycles?
(6) Was there occurrence or absence of amenorrheaafter embolization?
(7) Has a decrease in dysmenorrhea and dyspareunia been observed after embolization?
(8) Do you feel anxious about the unpredictable onset or duration of your periods after embolization?
(9) Do you feel anxious about traveling and have you decreased your physical activities after embolization?
(10) Do you feel irritable and have trouble sleeping or have you reduced the time you spend exercising or in other physical activities after the embolization?
(11) Do you find it difficult to carry out your normal activities and social activities after the embolization?
(12) Do you feel that you do not have control over your health after the embolization or that the situation is unchanged?
(13) Do you feel that your sexual activity has increased or decreased after uterine embolization?
(14) Please refer to how satisfactory is the condition after embolization (pain, infection).
(15) Have you ever felt pressure or tightness in your pelvic area over the last months?
(16) Did the presence of symptoms reduce your libido or did it prevent you from having sex?

### 2.3. Statistical Analysis

Statistical analysis of the data was performed using the Statistical Package for the So-cial Sciences (SPSS), version 19.0 (SPSS, Inc., Chicago, IL, USA). The normality of quantitative variables was tested with Kolmogorov–Sminrov test. Normally distributed quantitative variables (i.e., age) were expressed as the mean ± standard deviation (SD), while non-normally distributed quantitative variables (i.e., follow-up time) were expressed as the median and range (1 min–max 14 months). Qualitative variables were expressed as absolute and relative frequencies (%). To assess the effect of embolization on patients’ symptoms the Mc Nemar’s test was used. All tests were two-tailed and statistical significance was considered for *p*-values less than 0.05.

## 3. Results

The technical success of the method was 100%, with the women that underwent the procedure declaring to be very satisfied. UAE was performed by transdermal catheterization of the femoral artery, followed by X-ray approach of the uterine artery and infusion of media such as PVA (polyvinyl alcohol) particles or acrylic gelatin microspheres. The goal was vascular deformation of the fibroids and their shrinkage. Its effectiveness reached 80 to 100% in reducing bleeding and 40 to 60% in compressive symptoms. The patients tolerated the therapeutic intervention very well. In 2% of women, mild symptoms were observed (abdominal pain with lower intensity compared with preprocedural pain, vomiting—vomiting, fever) lasting for a few hours (up to 48 h), without further consequences.

**Figure 1:**
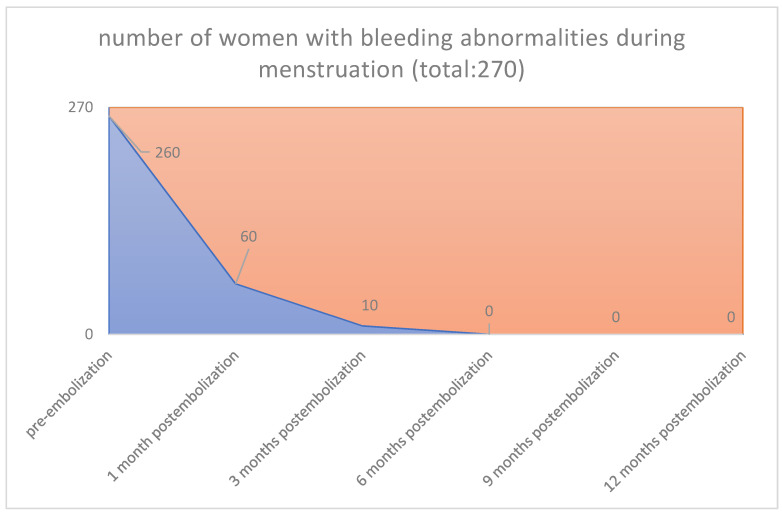
**Bleeding abnormalities: Pre-embolization**: Bleeding abnormalities during menstruation was reported in about 260 women out of a total 270 women. **1 Month postembolization** from 260 women 60 report bleeding abnormalities during menstruation. **3 Months postembolization** from 260 women only 10 participants report bleeding abnormalities during menstruation. **6 Months postembolization** from 260 women 0 participants report bleeding abnormalities during menstruation. **9 Months postembolization** from 260 women 0 participants report bleeding abnormalities during menstruation. **12 Months postembolization** from 260 women 0 participants report bleeding abnormalities during menstruation.

**Figure 2:**
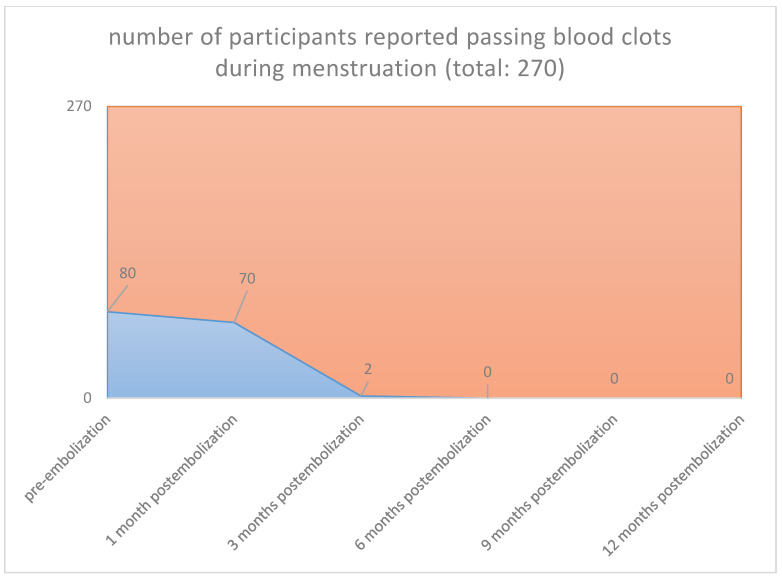
**Blood clots: Pre-embolization 80 from 270** participants reported passing blood clots during menstruation. **1 Month postembolization 70 from 80** participants reported passing blood clots during menstruation. **3 Months postembolization** only 2 from participants reported passing blood clots postembolization, which they have submucosal fibroma and they under-went in fractional curettage three months postembolization. **6 Months postembolization 0 from 80** participants reported passing blood clots during menstruation. **9 Months postembolization 0 from 80** participants reported passing blood clots during menstruation. **12 Months postembolization 0 from 80** participants reported passing blood clots during menstruation.

**Figure 3:**
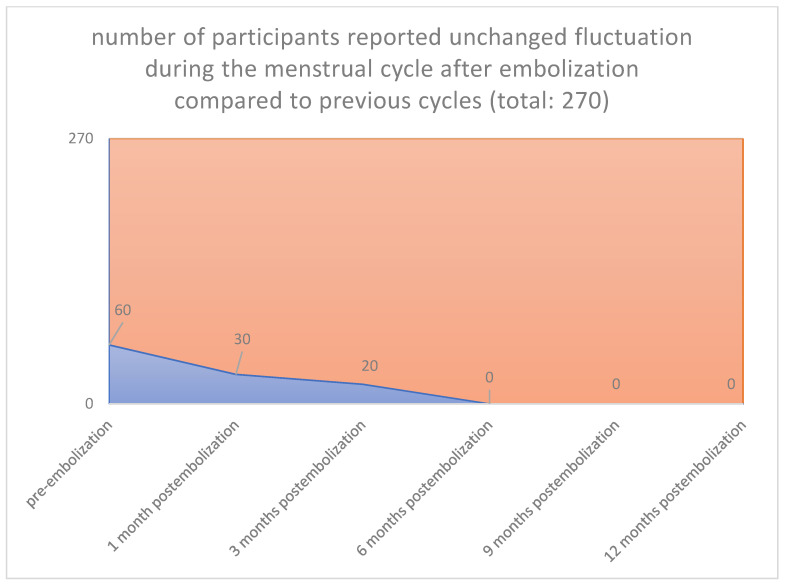
**The fluctuation during the menstrual cycle: Pre-embolization 60 from 270** participants reported fluctuation during the menstrual cycle. **1 Month 30 from 60 participants** reported unchanged fluctuation during the menstrual cycle. **3 Months 20 from 60 participants** reported fluctuation during the menstrual cycle. **6 Months 0 from 60 participants** reported unchanged, but significantly reduced fluctuation during the menstrual cycle. **9 Months 0 from 60 participants** reported unchanged flatulence, but significantly reduced pelvic floor heaviness, frequent urination during the menstruation’s day. **12 Months0 from 60 participants** reported unchanged flatulence, but signifi-cantly reduced fluctuation during the menstrual cycle.

**Figure 4:**
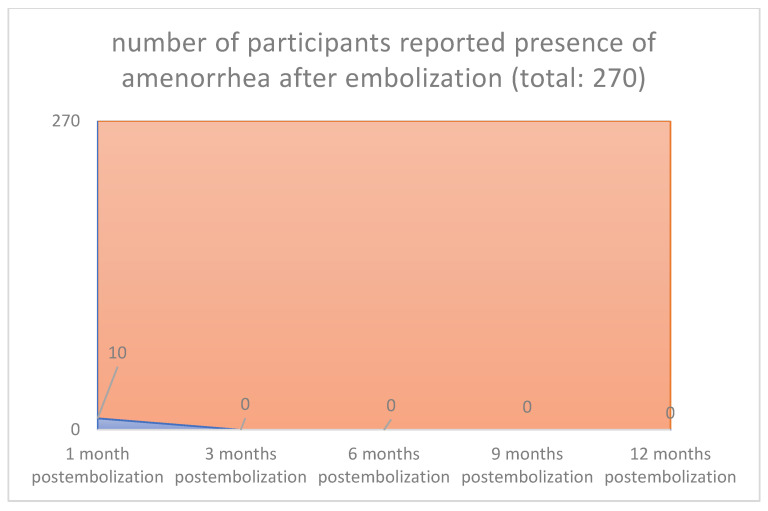
**Presence or absence of amenorrhea: 1 Month 10 from 270 participants** reported presence of amenorrhea after em-bolization aged greater than 45 years. **3 Months 0 from 10 participants** reported presence of amenorrhea after embolization. 6 Months 0 from 10 participants reported presence of amenorrhea after embolization. **9 Months 0 from 10 participants** reported presence of amenorrhea after embolization. **12 Months 0 from 10 participants** reported presence of amenorrhea after embolization.

**Figure 5:**
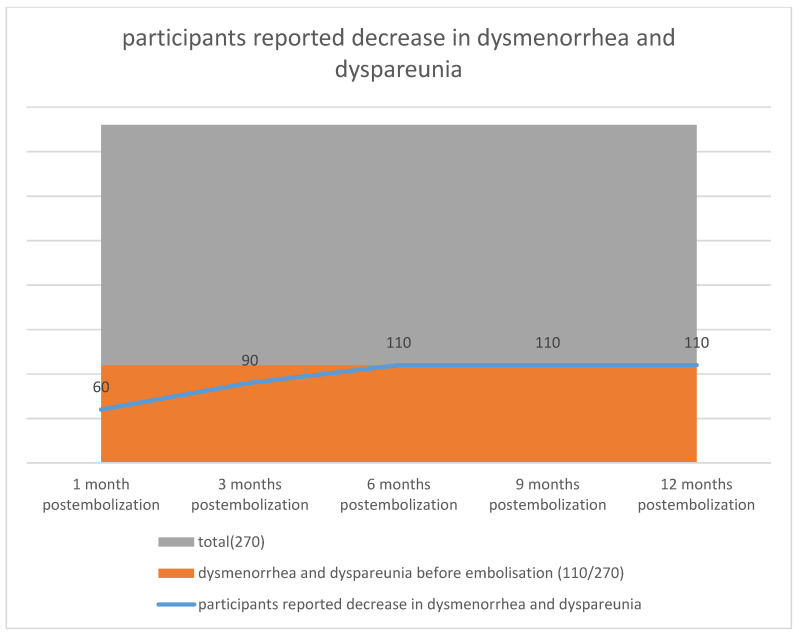
**Dysmenorrhea and dyspareunia: Pre-embolization 110 from 270 participants** reported dysmenorrhea and dyspareunia. **1 Month 60 from 110 participants** reported **decrease** in dysmenorrhea and dyspareunia. 3 Months 90 from 110 participants reported **decrease** in dysmenorrhea and dyspareunia. **6 Months 110 from 110 participants** reported **decrease** in dysmenorrhea and dyspareunia. **9 Months 110 from 110 participants** reported **decrease** in dysmenorrhea and dyspareunia. **12 Months 110 from 110 participants** reported **decrease** in dysmenorrhea and dyspareunia.

**Figure 6:**
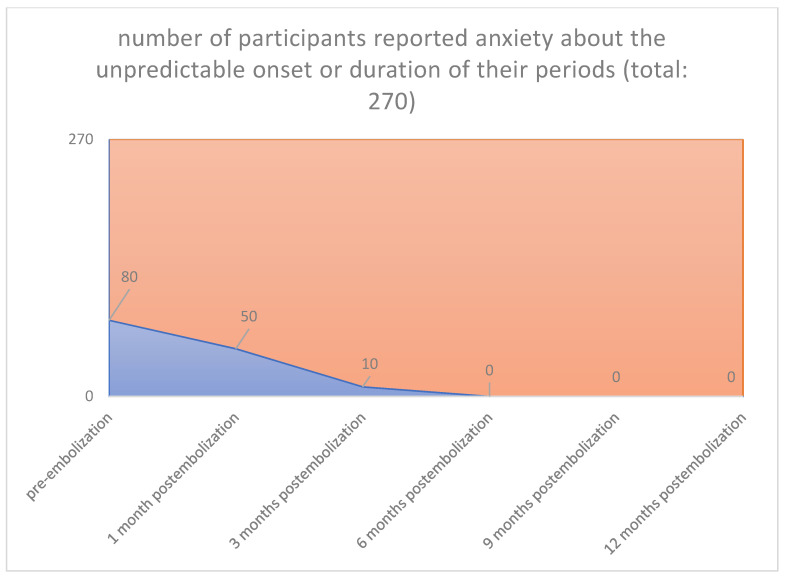
**Anxious about the unpredictable onset or duration of your periods: Pre-embolization 80 from 270 participants** reported anxiety about the unpre-dictable onset or duration of their periods. **1 Month 50 from 80 participants** reported anxiety about the unpredictable onset or duration of their periods. **3 Months 10 from 80 participants** reported anxiety about the unpredictable onset or duration of their periods. **6 Months 0 from 80 participants** reported anxiety about the unpredictable onset or duration of their periods. **9 Months 0 from 80 participants** reported anxiety about the unpredictable onset or duration of their periods. **12 Months 0 from 80 participants** reported anxiety about the unpredictable onset or duration of their periods.

In all cases, the clinical symptoms subsided. With the magnetic tomography of the first month, complete elimination of the vascularity of the fibroids was documented. Sequential magnetic resonance imaging examined the qualitative characteristics of fibroid degeneration (by volume) and their shrinkage. Shrinkage had already been studied at 3 months for the 90% of the participants and was 17% in the 3rd month, 55% in the 6th and 73% in the 12th month. According to our results, which are reported in our previous publications [26,27,28,29,30,31,32,33,34,35,36], we noticed no changes levels of examined laboratory parameters before and after one-year post-procedure. Reduction in symptoms was expected to reach 90%. Specifically, menorrhagia reduced in 76.92% in the first month and 96.15% in the third month postinterventional, respectively, and in all cases from sixth month until the end of follow up period (260/270 [96.3%] vs. 0/270 [0%], *p* < 0.001), (80/270 [29.6%] vs. 0/270 [0%], *p* < 0.001(Figure 1 and Figure 2), respectively. The compressive symptoms (flatulence, pelvic weight and frequency urination during the day, the nighttime hours) were reduced by 81–100% (90/270 [33.3%] vs. 0/270 [0%], *p* < 0.001), (60/270 [22.2%] vs. 0/270 [0%], *p* < 0.001), respectively. No patient had permanent amenorrhea after the procedure. However, in a small number of participants older than 45 years, a postinterventional transient amenorrhea was observed in the first 3 months (60/270 [22.2%] vs. 0/270 [0%], *p* < 0.001) (20/270 vs. 0/270 [0%], *p* < 0.001) (Figure 3 and Figure 4), respectively. It was notable a decrease in dysmenorrhea and dyspareunia, absence of feeling anxious about the unpredictable onset or duration of periods, willing about traveling and improving of physical activities, absence feeling irritable in association with difficulties in sleeping and reduction in the time spending for exercise or for other physical activities after the embolization110/270 [40.7%] vs. 0/270 [0%], *p* < 0.001) (80/270 [29.6%] vs. 0/270 [0%], *p* < 0.001) (Figure 5 and Figure 6), respectively. No difficulties were noticed to carry out with normal and social activities (75/270 [27.8%] vs. 0/270 [0%], *p* < 0.001) (85/270 [31.5%] vs. 0/270 [0%], *p* < 0.001) (95/270 [35.2%] vs. 0/270 [0%], *p* < 0.001) (105/270 [38.9%] vs. 0/270 [0%], *p* < 0.001) (Parameters 9–12 from the mentioned questionnaire), respectively.

**Figure 7:**
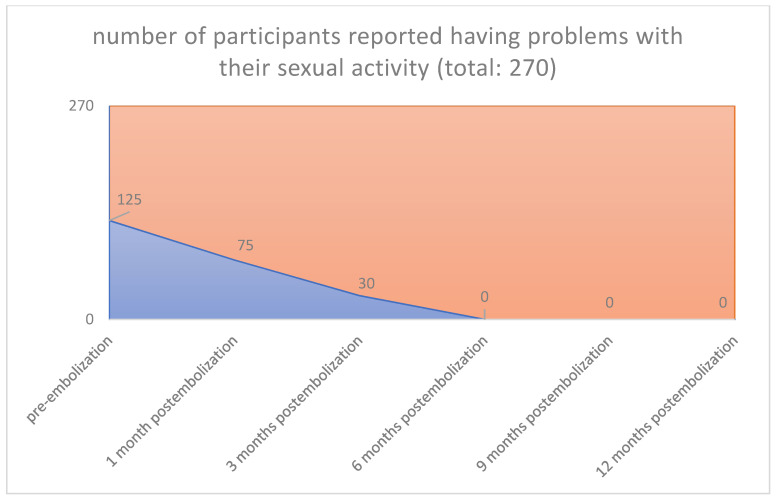
Sexual activity: Pre-embolization 125 from 270 participants reported having problems with their sexual activity. 1 Month 75 from 125 participants reported having problems with their sexual activity. 3 Months 30 from 125 participants reported having problems with their sexual activity. 6 Months 0 from 125 participants reported having problems with their sexual activity. 9 Months 0 from 125 participants reported having problems with their sexual activity. 12 Months 0 from 125 participants reported having problems with their sexual activity.

**Figure 8:**
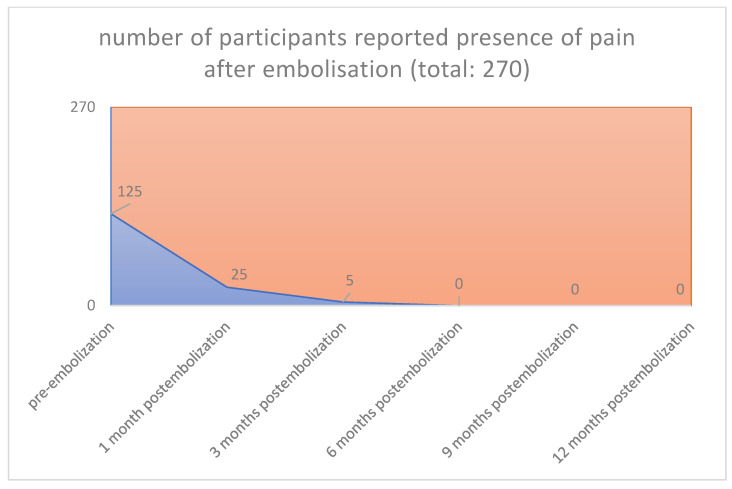
**Pain, infection:. Pre-embolization 125 from 270 participants** reported presence of pain. **1 Month 25 from 125 participants** reported presence of pain, infection. **3 Months 5 from 125 participants** reported presence of pain. **6 Months 0 from 125 participants** reported presence of pain. **9 Months 0 from 125 participants** reported presence of pain. **12 Months 0 from 125 participants** reported presence of pain.

**Figure 9:**
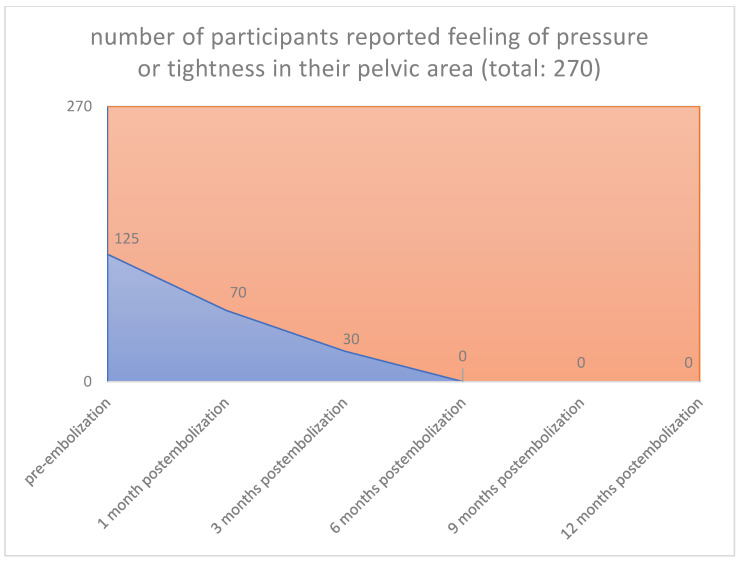
**Pressure or tightness in pelvic area: Pre-embolization 125 from 270 participants** reported feeling of pressure or tightness in their pelvic area. **1 Month 70 from 125 participants** reported feeling of pressure or tightness in their pelvic area. **3 Months 30 from 125** felt pressure or tightness in their pelvic area. **6 Months 0 from 125** felt pressure or tightness in their pelvic area. 9 Months 0 from 125 felt pressure or tightness in their pelvic area. **12 Months 0 from 125** felt pressure or tightness in their pelvic area.

**Figure 10:**
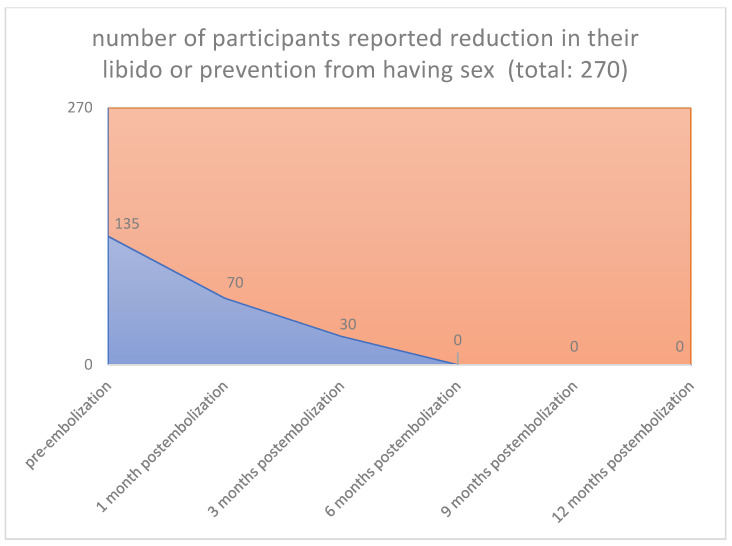
**Libido: Pre-embolization 135 from 270 participants** reported reduction in their libido or prevention from having sex. **1 Month 70 from 135 participants** reported reduction in their libido or prevention from having sex. **3 Months 30 from 135 participants** reported reduction in their libido or prevention from having sex. **6 Months 0 from 135 participants** reported reduction in their libido or prevention from having sex. **9 Months 0 from 135 participants** reported reduction in their libido or pre-vention from having sex. **12 Months 0 from 135 participants** reported reduction in their libido or prevention from having sex.

An increase of sexual activity, a decrease in pressure or tightness in the pelvic area, absence of infection and pain, and an increase of libido over the last months after uterine embolization were also observed (125/270 [46.3%] vs. 0/270 [0%], *p* < 0.001) (125/270 [46.3%] vs. 0/270 [0%], *p* < 0.001) (115/270 [42.6%] vs. 0/270 [0%], *p* < 0.001) (135/270 [50.0%] vs. 0/270 [0%], *p* < 0.001) (Figure 7, Figure 8, Figure 9 and Figure 10), respectively.

Fibroid size exhibited a mean reduction of 17%, 55%, and 75% over a follow up period of 3, 6, and 12 months, respectively. The uterine size was not appeared to be a decisive factor, since remission of symptoms was observed in the same percentage of patients with a uterus larger than 24 weeks. No major complications (including allergic reaction, persistent prolonged pain, urinary tract infection, endometritis, femoral nerve injury, vascular injury, drug reaction, deep vein thrombosis, and pulmonary embolism) were noticed.

## 4. Discussion

Uterine artery embolization (UAE) is indicated in those cases of symptomatic fibroids in which other pharmacological and surgical treatments are either contraindicated, fail, or are not desired by the patient. It is an alternative to hysterectomy and is performed by specialized interventional radiologists [4,5,6,7,8,9].

Uterine fibroid embolization is a minimally bloody procedure that has been clinically proven to reduce the main symptoms of fibroids, such as pelvic pain and pressure, excessive, prolonged bleeding, frequent urination, and can effectively help improve the quality of life of postpartum women [10,11,12]. Although the embolization is performed by an invasive radiologist, while the examination before the operation by a gynecologist, cooperation between both specialties is necessary. Since the method is relatively new and involves other specialties besides gynecology, many gynecologists do not recommend it as an alternative technique in the treatment of fibroids (since its final results have not yet been investigated). Nevertheless, women are informed of this method and often seek further information. The most ideal candidates for UAE are perimenopausal women with symptomatic fibroids who do not want to have children and have been tested with imaging tests and endometrial biopsy (so that the existence of malignancy has been ruled out) [24,25,26]. It should be noted that the technical success of the method was 100% in our patients, with the women that underwent the procedure declaring to be very satisfied.

In terms of exclusion criteria, although the uterine fibroid size generally is not an absolute contraindication, sometimes it was necessary to perform a repeat intervention. Large fibroids (> 15cm) are expected to shrink, but their final size can still cause compressive symptoms, so they are a relative contraindication. Other conditions to consider are the presence of submucosal or multifocal fibroids, which are best endoscopically since their necrosis can lead to their elimination with the late onset of bleeding and pain [24,25,26,27]. The coexistence of an active infection that can flare up again with ischemia, a history of radiotherapy that has already reduced uterine perfusion, and renal failure due to the toxic action of the shader represent contraindications.

Allergic reactions can be prevented with appropriate preoperative treatment [24,25,26,27]. UAE concerns those cases of symptomatic fibroids in which other drug and surgical treatments are either contraindicated, fail, or are not desired by the patient [24,25,26,27]. It is an alternative to hysterectomy and is performed by specialized invasive radiologists. Catheter uterine artery embolization for the treatment of symptomatic uterine fibroids is a non-amputating and almost bloodless invasive interventional treatment method with alternative hysterectomy, which keeps the female uterus intact and functional. The incidence of UAE complications is very low [28,29,30,31,32,33,34].

Complications are generally related to either catheterization or the effects of uterine ischemia that can cause fibroid necrosis with a septic appearance and their elimination. Finally, embolization of other organs (especially of the ovaries) may occur. Deaths reported after embolization are extremely rare (1:1600) and are mainly related to pulmonary embolism, which can occur from the effect of dead tissue on the activation of the coagulation mechanism and infection [30,31,32,33,34].

Complications of catheterization are rare (<1%), such as hematoma, contrast allergy, and pseudoaneurysm or vascular segregation. Ovarian failure and the effects on fertility and pregnancy are unknown.

The recurrence of fibroids reaches 10%, but is believed to be due to an increase in the size of the old implantable and adenomyosis. The main failure factor was not the initial size of the fibroid, but the failure to shrink it below 30% of the original size [30,31,32,33,34].

According to our previously published studies [35,36,37], it was confirmed in accordance with the international literature that the UAE has no negative influence on ovarian function. The best evidence for this is that postprocedural AMH levels were identical to related preprocedural hormonal levels. The occurrence of amenorrhea in women younger than 45 years was 0%, and we have noticed transient amenorrhea only in the first 3 months post-procedure in 0.6%. The reported amenorrhea rate in the international literature after uterine fibroids embolization is reported by 8.1% [38,39]. The infection parameters had an uneventful postprocedural course.

It should be noted that there are no long-term data, although studies with follow-up of up to 32 months show that women remain highly satisfied [30,31,32,33,34]. Compared to works in the previously published literature [40,41,42,43,44,45,46,47,48], the life quality parameters which were exanimated based on anonymous questionnaires confirm a satisfactory high level. The main symptoms which have led the premenopausal women to choose this conservative method including irregular vaginal bleeding massive symptoms to pelvic organs, minor sexually activity due to massive symptoms, and pain were satisfactory eliminated. All participants were very satisfied and they recommended the UAE to their friendly environment.
